# Exploring the Diverse Morphology of Porous Poly(Lactic Acid) Fibers for Developing Long-Term Controlled Antibiotic Delivery Systems

**DOI:** 10.3390/pharmaceutics14061272

**Published:** 2022-06-15

**Authors:** Kwon Ho Seo, Kyung Eun Lee, Meltem Yanilmaz, Juran Kim

**Affiliations:** 1 Advanced Textile R&D Department, Korea Institute of Industrial Technology (KITECH), Ansan 15588, Korea; tjrnjsgh@kitech.re.kr; 2 Department of Mechanical Engineering, Inha University, 100 Inharo, Incheon 22212, Korea; bfmec@inha.ac.kr; 3 Department of Textile Engineering, Istanbul Technical University, Istanbul 34467, Turkey

**Keywords:** antibacterial assay, porous poly(lactic acid) fibers, solvent-polymer system, aminoglycoside derivatives, controlled drug release, drug delivery

## Abstract

In this study, we aimed to explore the morphologies of porous poly(lactic acid) (PLA) fibers through liquid–liquid phase separation, and investigate the relationship among pore formation, physical properties, and antibacterial activities of the fibers for identifying their potential as drug delivery carriers. Antibacterial activities of gentamicin-, kanamycin-, and amikacin-loaded PLA fibers against *E. coli* and *S. epidermidis* were evaluated. The antibacterial activity of drugs against *E. coli* showed the following profile: gentamicin > amikacin > kanamycin; however, *S. epidermidis* growth was almost completely inhibited immediately after the administration of all three drugs. The efficiency of gentamicin can be attributed to the electrostatic interactions between the positively and negatively charged antibiotic and bacterial cell membrane, respectively. Furthermore, gentamicin-loaded porous PLA fibers were evaluated as drug delivery systems. The cumulative amount of gentamicin in porous PLA nanofibers was considerably higher than that in other PLA fibers for 168 h, followed by 7:3 PLA > 6:4 PLA > 5:5 PLA > non-porous PLA. The 7:3 PLA fibers were projected to be ideal drug carrier candidates for controlled antibiotic release in delivery systems owing to their interconnected internal structure and the largest surface area (55.61 m^2^ g^−1^), pore size (42.19 nm), and pore volume (12.78 cm^3^ g^−1^).

## 1. Introduction

Aminoglycoside derivatives (ADs) have been reported as broad-range antibiotics that can treat lethal infections by inhibiting various Gram-positive and Gram-negative bacteria [1,2]. The core structure of ADs includes a variety of amino groups, hydroxyl groups, and side chains (substituents) that play a critical role in their antibacterial activity. They are the key elements that bind to the A-site on the 16S ribosomal RNA of the 30S subunit in the target bacterial membrane, resulting in protein mistranslation [3]. The class of gentamicin (most frequently used AD) includes a 4, 6-substituted 2-deoxystreptamine and two hexoses with additional carbon side chains [1,4,5]. The kanamycin class contains 4,6-substituted 2-deoxystreptamin derivatives coupled with 3-aminoglucose and 2-amino- or 2,6-diamino-glucose [6]. Amikacin, an amino cyclitol glycoside and one of the most effective semisynthetic aminoglycosides, can be synthesized by acylation at the N-1 position of a 4-amino-2-hydroxybutyryl group of kanamycin [7].

There is a need for specific drug carriers that can protect and deliver these ADs effectively. Carriers are generally used in the form of capsules, porous fibers, and core-shells as they allow the ADs to remain unaltered, survive severe body conditions, and release their contents at the desired time. Among these carriers, electrospun fibers have been studied for many decades as no additional chemical reactions are required during electrospinning and there are no alterations to the chemical structure of antibiotics [8]. Electrospinning produces fibers with varied morphologies and high surface-to-volume ratios by controlling processing variables, such as flow rate, voltage, distance between a nozzle and a collector, nozzle types, humidity, concentrations, and polymer–solvent selection [9]. An additional benefit of this method is the ability to induce porosity in fibers, which provides additional sites for antibiotic adsorption [10]. Increased porosity of fibers results in increased surface area and volume, leading to improved effectiveness of carrying antibiotics [11]. Additionally, the morphology of porous fibers influences the release characteristics of antibiotics, including diffusion, release periods, degradation, and bursting release at the initial stage [12].

**Figure 1:**
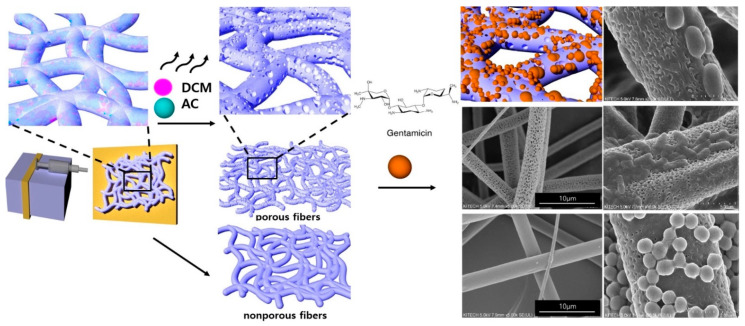
Scheme of pore formation in electrospun porous and nonporous PLA fibers by liquid–liquid phase separation method and antibacterial PLA microfibers.

Three types of phase separation methods to produce porous fibers have been reported thus far: first, thermally induced phase separation, which involves the evaporation of a volatile solvent at different temperatures [13]; second, non-solvent induced phase separation, in which precipitation occurs by absorption/penetration of a non-solvent, like water, from the vapor phase into fiber jet; third, vapor induced phase separation due to the difference in vapor pressure of solvents [14]. Due to the accelerated evaporation of the solvent during electrospinning, as phase separation occurs, polymer-deficient regions transform into pores or voids [15,16]. The mechanism of pore formation by phase separation and the development of antibaterial PLA microfibers are shown in Figure 1.

Although many researchers have successfully developed various types of porous nanofibers by different methods, including electrospinning, there is limited research on porous poly (lactic acid) (PLA) microfiber fabrication depending on polymer–solvent systems and fiber structure–antibacterial property relationship. Furthermore, the time design of controlled antibiotic release from various types of porous PLA microfibers and the underlying mechanisms have seldom been reported [12]. Additionally, little is known about the effects of various porous microfiber morphologies on long-term release characteristics, such as large-burst drug release, uncontrolled drug release duration, and incomplete drug release [12,17].

Therefore, in this study, a variety of porous surfaces were developed using polymer–solvent systems to enhance surface physical properties. Antibacterial properties of ADs and their release profiles were correlated to the morphologies of the fibers. This investigation will provide a better and comprehensive understanding of porous microfiber formation during electrospinning and the fiber morphology–antibacterial property relationship.

## 2. Materials and Methods

### 2.1. Materials

PLA granules were purchased from Goodfellow (Goodfellow Cambridge Limited, Huntingdon, UK). Gram-negative strain *Escherichia coli* (*E. coli*, KCTC 1039) and Gram-positive strain *Staphylococcus epidermidis* (*S. epidermidis,* KCTC 13172) were obtained from Korean Collection for Type Cultures (KCTC, Jeongyep-si, Korea). Kanamycin disulfate salt from *Streptomyces kanamyceticus*, gentamicin sulfate, amikacin, phosphate-buffered saline (PBS, pH 7.4), sodium chloride, tryptic soy broth, LB Broth, dichloromethane (DCM), acetone (AC), chloroform, ethyl alcohol (EtOH), ninhydrin, acetic acid, and osmium tetroxide (4% *w*/*v*) were purchased from Sigma-Aldrich (St. Louis, MO, USA). Tryptic soy agar and MacConkey sorbitol agar were purchased from Becton Dickinson (BD Difco™, Sparks, MD, USA).

### 2.2. Preparation of Electrospun Porous and Non-Porous PLA Fibers

For porous PLA fibers, a 10% (*w*/*v*) PLA solution with different volume ratios of DCM and AC, i.e., 5:5 (5:5 PLA), 6:4 (6:4 PLA), and 7:3 (7:3 PLA), were prepared, and then electrospun at a feeding rate of 3 mL h^−1^ at 18 kV. For non-porous PLA fibers, a 15% (*w*/*v*) PLA solution with a 7:3 volume ratio of chloroform and EtOH was prepared, and then electrospun at a feeding rate of 1.5 mL h^−1^ at 18 kV. The distance between the nozzle tip and roller collector was 15 cm. All PLA fibers were treated with oxygen plasma at 200 W and 160 sccm for 5 min to increase wettability using a plasma system (Femto Science, Hwaseong, Korea).

### 2.3. Preparation of AD-Loaded PLA Fibers

Antibiotic solutions of kanamycin disulfate salt, gentamicin, or amikacin were prepared at a concentration of 512 μL mL^−1^. Porous and non-porous PLA fibers were soaked in the solution and placed in a shaking incubator at 25 °C and 5 relative centrifugal force (RCF) for 24 h. Then, the PLA fibers were dried for 2 h at room temperature.

### 2.4. Characteristics and Morphology of Porous and Non-Porous PLA Fibers

The morphologies of porous and non-porous PLA fibers prepared from different solvent systems were observed by a scanning electron microscope (SEM, Hitachi, Tokyo, Japan) using the Nawhoo imaging software (Iworks 2.0, Suwon, Korea) (N ≥ 100). To measure the surface topologies of porous PLA fibers, an atomic force microscope (AFM, Park system, Yongin, Korea) was used. First, the fibers were mounted onto carbon tape, following which the size and depth of the pores were analyzed using Park system XEI program 5.2.0 (Park system, Yongin, Korea). The N_2_ adsorption analyses of non-porous PLA, porous 5:5, 6:4, and 7:3 PLA fibers were conducted using BELSORP-Mini II (MicrotracBEL, Osaka, Japan) at 77 K. The specific surface area, average pore size, and total pore volume were calculated from the adsorption curve according to the Brunauer–Emmett–Teller (BET) and Barrett-Joyner-Halenda (BJH) methods in the *p*/*p*_0_ range of 0.0−1.0 using the BELSORP analysis software. The viscosities of fibers were measured at 25 °C using a Brookfield RVT rheometer (Toronto, ON, Canada). A thermogravimetric analyzer (TA Instruments, Delaware, USA) was used to evaluate the thermal stability of the gentamicin-loaded 7:3 PLA fibers in a N_2_ atmosphere with a heating rate of 20 °C min^−1^ from 25 to 500 °C.

### 2.5. Quantitative Antibacterial Growth Curves Assay of AD-Loaded PLA Fibers

Antibacterial growth of AD-loaded PLA fibers and free ADs was evaluated by generating kinetic growth curves using the measured OD_600_ values. Each PLA sample was prepared (20 × 20 mm) and added to 10 mL of a bacterial suspension of *E. coli* and *S. epidermidis* at the concentrations of 4.22 × 10^6^ and 1.72 × 10^7^ CFU mL^−1^, respectively. They were then cultured for 72 h in a shaking incubator at 150 rpm and 37 °C. Following this, the values of OD_600_ were measured and used to plot the growth kinetic curves. The inhibition % was calculated by using Equation (1):

(1) $$Inhibition=\left(1-\frac{OD600\;of\;the\;treated\;sample\;at\;incubation\;time}{OD600\;of\;the\;PLA\;control\;at\;incubation\;time}\right)\times 100$$

To observe the adhesion of bacterial cells on AD-loaded 7:3 PLA after 72 h of incubation, the cells were fixed using osmium tetroxide vapor (2% *w*/*v*) for 24 h and analyzed by SEM (Hitachi, Tokyo, Japan). The statistical difference between groups for antibacterial activities of 7:3 AD-loaded PLA fibers was calculated using two-way ANOVA (Tukey’s multiple comparison post hoc test). The level of statistical significance was defined as *p* < 0.05.

### 2.6. In Vitro Release Assay of Gentamicin-Loaded PLA Fibers

In vitro release assay of gentamicin-loaded PLA fibers was conducted using a quantitative colorimetric assay to compare the amount of released gentamicin and the release pattern from porous and non-porous PLA fibers for 7 d. Gentamicin-loaded porous PLA fibers (5:5, 6:4, 7:3 PLA, and non-porous) were prepared. First, each gentamicin-loaded PLA sample was incubated in 3 mL of PBS at 37 °C for 7 d. One mL of this solution was transferred to a 5 mL vial. Additionally, 1 mL of pure PBS was added to the gentamicin-loaded PLA in the original PBS sample. Next, 0.3 mL of ninhydrin solution (5 mg mL^−1^) was added to the 5 mL vial and incubated at 95 °C in a water bath for 15 min. After cooling down, OD_405_ of the vial was measured using a microplate reader (Allsheng, Hangzhou, China) and the average values were calculated. All experiments were performed in triplicate.

## 3. Results

**Figure 2:**
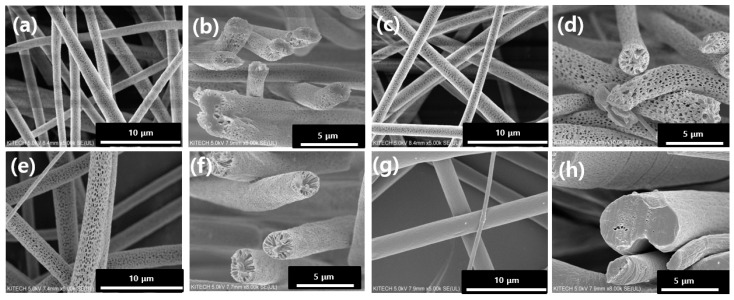
SEM images of porous and non-porous PLA microfibers in different polymer–solvent systems: (**a**) 5:5 PLA, (**b**) the cross-section of 5:5 PLA, (**c**) 6:4 PLA, (**d**) the cross-section of 6:4 PLA, (**e**) 7:3 PLA, (**f**) the cross-section of 7:3 PLA, (**g**) non-porous PLA, and (**h**) the cross-section of non-porous PLA.

Non-porous and porous PLA fibers (5:5, 6:4, and 7:3 PLA) were fabricated by electrospinning. These PLA fibers were coded as shown in Table 1. Figure 2 depicts the surface morphology of the electrospun PLA fibers and their cross-sectional images. Non-porous PLA fibers exhibited a smooth surface with an average fiber diameter of 2.28 µm. The increased amount of DCM in polymer–solvent systems showed a corresponding increase in the average fiber diameter from 1.87 to 2.43 µm as shown in Table 1. As the DCM content increased up to 70%, the fiber diameter increased to 2.43 µm.

Pore formation in PLA microfibers was caused due to phase separation during electrospinning, which occurs when the polymer solution is released from the nozzle into a solvent-free environment. The DCM at the surface of the polymer–solvent jet evaporates rapidly, whereas AC within the jet diffuses outward concurrently. The rapid vaporization of polymer–solvent induces phase separation to form polymer-rich and polymer-deficient phases [14,18]. The concentrated polymer-rich phase solidifies to form the matrix, whereas the polymer-deficient phase creates voids or pores [19]. This phenomenon could be due to the fact that the *p_v_* and viscosity of DCM are higher (approximately 58.1 kPa and 0.449 mPa·s at RT) compared to those of AC (approximately 30 kPa and 0.308 mPa·s at RT) [20]. DCM is expected to evaporate relatively quickly during the electrospinning process, leaving behind AC (polymer-rich phase) [21]. This hypothesis is supported by the observations in Figure 2, which shows that the number of pores increases as the DCM content increases to 70% by volume.

Porous PLA fibers display varied internal structures, such as pore depth, size, and volume, depending on the polymer–solvent systems. This was confirmed by changing the solvent ratio of DCM:AC. The different polymer–solvent systems for fabricating porous PLA fibers led to the formation of an interpenetrating network structure between the PLA polymer-rich (PLA in AC) and polymer-deficient phases (PLA in DCM). The liquid–liquid phase separation occurred due to the coarsening effect, yielding nano- or micro-sized pores in PLA fibers [22,23].

**Figure 3:**
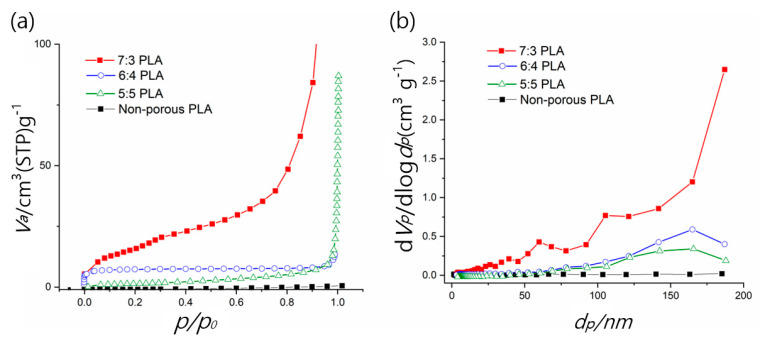
(**a**) N_2_ adsorption isotherms and (**b**) pore size distribution (BJH) plots of non-porous and porous PLA fibers (5:5, 6:4, and 7:3 PLA).

Figure 3a presents the N_2_ adsorption isotherms of non-porous and porous PLAs (5:5, 6:4, and 7:3 PLAs). The 7:3 PLA fibers showed an increase in specific surface area (55.61 m^2^ g^−1^), which is up to 1853 times higher than that of non-porous PLA fibers (0.030 m^2^ g^−1^). From the BJH plot analysis, we observed that when DCM content in polymer–solvent systems is increased up to 70%, the total pore volume and average pore size increase to 12.78 cm^3^ g^−1^ and 42.19 nm, respectively (Figure 3b and Table 1). These results demonstrated the successful formation of various porous PLA fibers with mesoporous structures. In this study, 7:3 PLA exhibited the largest surface area and average pore size and volume, as depicted in Table 1.

**Figure 4:**
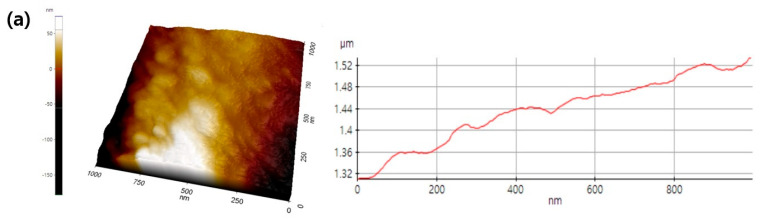

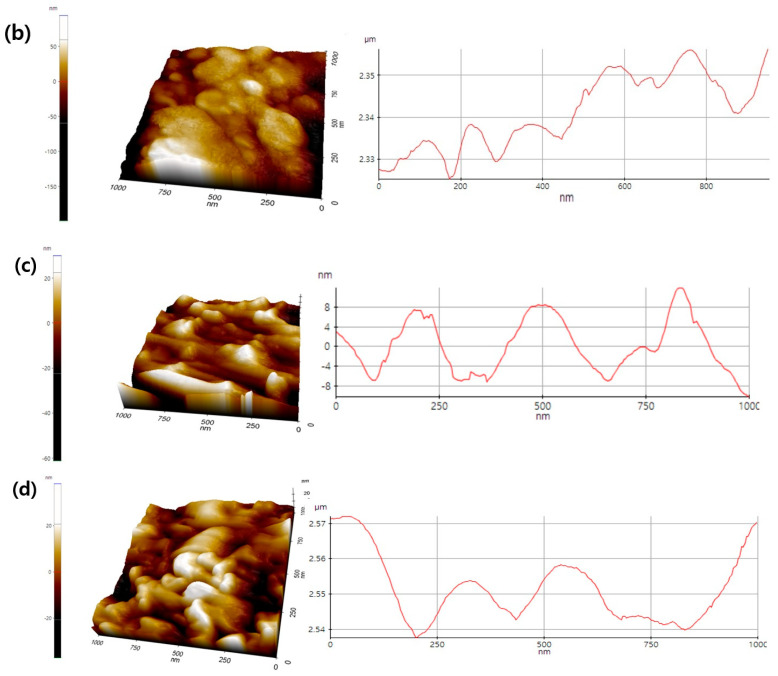
AFM images of PLA fibers; (**a**) non-porous PLA, (**b**) 5:5, (**c**) 6:4, and (**d**) 7:3 PLA.

To investigate the effect of polymer–solvent systems on roughness or pore depth, we analyzed AFM images and discovered that nano-sized pores interconnected regularly on the surface of PLA fibers, as shown in Figure 4. The measurements of roughness of all PLA fibers and corresponding mean values are depicted in Figure 4. The mean roughness value of non-porous PLA fibers was approximately less than 5 nm, while that of porous 7:3 PLA increased over 100 nm. As observed in the SEM images and BET analysis, the pore depth and surface area in 7:3 PLA had a high magnitude. Phaechamud and Chrtrattha have reported that high viscosity liquids promote the formation of high pore sizes [24]. Moreover, the increased viscosity results in increased coarsening time, leading to the formation of large pores and interpenetrating network structures of PLA fibers [25]. In Table 1, 7:3 PLA had the highest viscosity of 252 mPa·s in the DCM:AC polymer–solvent systems, the interpenetrating network structure with the largest pore size and depth, as shown in Figure 4d.

**Figure 5:**
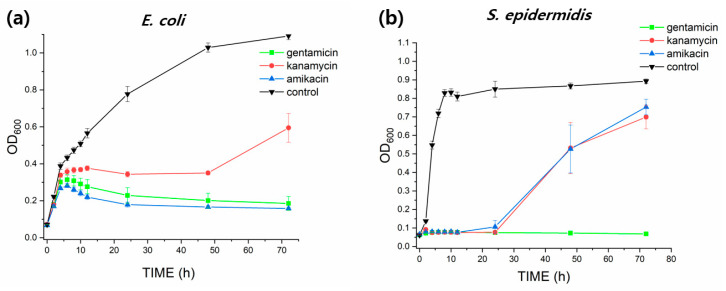

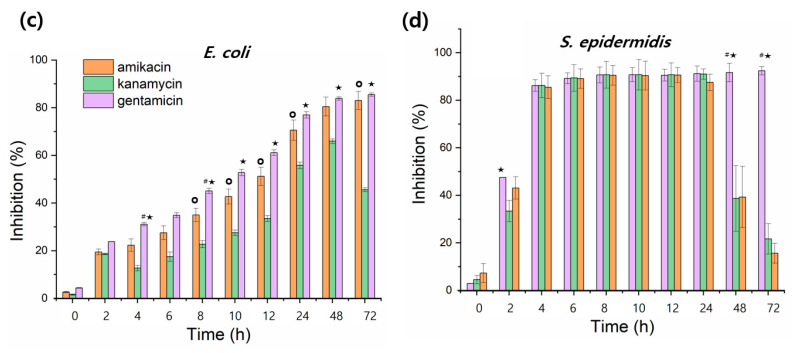
Antibacterial kinetic growth curves and inhibition (%) of non-porous PLA (control) and AD-loaded porous 7:3 PLA against (**a**,**c**) *E. coli* and (**b**,**d**) *S. epidermidis*; two-way ANOVA: **^o^** *p* < 0.05 amikacin versus kanamycin; ^★^ *p* < 0.05 kanamucin versus gentamicin; # *p* < 0.05 amikacin versus gentamicin.

From previous analyses, we know that 7:3 PLA had the largest surface area and average pore size and volume. This enabled 7:3 PLA to carry more antibiotics for a long period of time. For subsequent experimentation, 7:3 PLA was selected and loaded with ADs, such as kanamycin, gentamicin, and amikacin. To quantitatively analyze the antibacterial activities of all the AD-loaded 7:3 PLA fibers against *E. coli* and *S. epidermidis*, kinetic growth curves were constructed using OD_600_ values measured at regular intervals for 72 h (Figure 5). The antibacterial activity against *E. coli* was as follows: gentamicin > amikacin > kanamycin. The growth of *S. epidermidis* was almost completely inhibited immediately after subjecting all three ADs to them, whereas *E. coli* required more time to resist their action during the 24 h. The inhibition of *E. coli* was increased with the utilization of gentamicin and amikacin during 72 h, resulting in 84% inhibition. For AD-loaded PLA samples against *S. epidermidis*, the OD_600_ values increased after 24 h of incubation from 0 to 0.7 except gentamicin, whereas gentamicin and amikacin against *E. coli* were decreased or constant near 0.17 during 72 h of incubation. The inhibition of *E. coli* was increased with the utilization of gentamicin and amikacin during 72 h, resulting in 84% inhibition. For gentamicin-, amikacin-, and kanamycin-loaded 7:3 PLAs were almost thoroughly restrained within 24 h over 90% inhibition. Gentamicin-loaded 7:3 PLA exhibited great antibacterial performance against *S. epidermidis* with over 93% of inhibition within 72 h. Table 2 compared inhibition (%) of AD-loaded porous 7:3 PLA and free ADs against *E. coli* and *S. epidermidis* after 72 h. For free ADs against *E. coli* and *S. epidermidis*, the antibacterial acitivities showed immediately within 1 h and then reached the highest inhibition percent at 12 h time treatment (Figure S1). After 72 h time treatment, antibacterial activities of free gentamicin, kanamycin, and amikacin were decreased to 53, 40, and 55% inhibition against *E. coli*, respectively; 47, 18, and 8% inhibition against *S. epidermidis*, respectively. This observation can be attributed to the difference in the peptidoglycan layers in the cell walls of *E. coli* and *S. epidermidis*. *S. epidermidis* has a thicker peptidoglycan layer and higher cross-linking degree in the cell wall as compared to that in *E. coli* [26]. To reach their molecular target, the antibiotics must first penetrate the cell membrane of bacteria. Therefore, the total inhibition of *S. epidermidis* required higher concentration of ADs than for that of *E. coli*.

Antibacterial activity is related to the electrostatic binding of the polycationic ADs to the negatively charged components of the bacterial membrane, such as phospholipids and teichoic acids in Gram-positive organisms and the phospholipids and lipopolysaccharide in Gram-negative organisms [27]. This binding leads to error-prone protein synthesis, allowing amino acids to assemble into incorrect polypeptides that subsequently cause damage to the cell membrane and other organelles [27,28]. Yang et al. have reported that electrostatic binding energy of ADs to the A-site of the bacterial membrane and *G_elec_* of gentamicin (−144.5 KJ mol^−1^) increased binding energy more effectively than kanamycin (−101 KJ mol^−1^) [29]. Another study has demonstrated that gentamicin (8.5 kcal mol^−1^) had higher binding energy than amikacin (4.9 kcal mol^−1^) during the simulated interaction of aminoglycosides with phosphatidylinositol monolayers [30]. Amikacin or kanamycin may produce low amounts of endotoxins, leading to a low occurrence of early death, whereas gentamicin may show the opposite effect [31].

**Figure 6:**
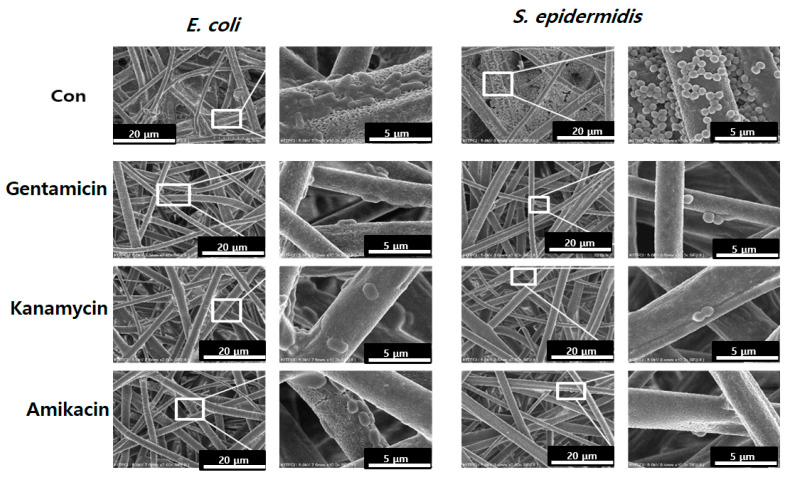
Bacterial adhesion on the AD-loaded 7:3 PLA fibers.

In Figure 6, SEM images depict the bacterial adhesion depending on porous 7:3 PLA with ADs or without ADs (control). In the 7:3 PLA control, high bacterial adhesion was observed, and the cells remained undamaged and were rod- and coccus-shaped with a smooth surface [32]. After 24 h of incubation, large colonies were observed on PLA fiber surfaces (control), whereas fewer bacteria appeared on the AD-loaded 7:3 PLA. Bacterial structural changes and decreases in the number of adhered bacteria were also observed in all AD-loaded 7:3 PLA. It has been reported that ADs show antibacterial activity within cells by introducing multiple errors in protein synthesis, leading to increased damage to the cytoplasmic membrane [28]. In our study, all AD-loaded PLA samples inhibited adhesion and colony formation in both *E. coli* and *S. epidermidis*.

**Figure 7:**
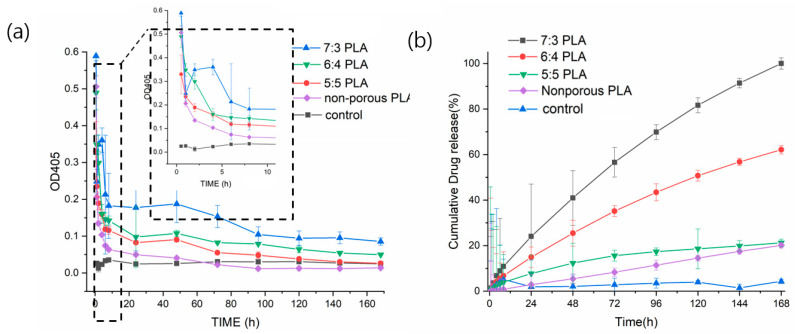
In vitro release profiles of gentamicin-loaded non-porous and porous 5:5, 6:4, and 7:3 PLA fibers for 7 d, (**a**) OD_405_ curves and (**b**) cumulative release profiles.

To investigate the underlying release patterns of antibiotics based on the types of porous PLA fibers, the release profile of gentamicin was analyzed. The standard curve of gentamicin concentration-OD_405_ obtained was y = 0.0221x + 0.00154 with adj. R^2^ = 0.951 (Figure S2). Gentamicin-loaded non-porous PLA was released quickly and decreased the release within 30 min, which was similar to the OD_405_ value of control (PLA fibers without gentamicin) in Figure 7a. It has been reported that drugs placed on or near the surface of fibers caused a burst release during the initial period [33]. However, the gentamicin-loaded 5:5, 6:4, and 7:3 PLAs showed a continuous release rate. Among these, gentamicin-loaded 7:3 PLA showed the highest drug release in the first 30 min (released gentamicin at a concentration of 26.61 µg mL^−1^) and displayed a bursting release pattern during the next 3–5 h. Furthermore, gentamicin-loaded 7:3 PLA displayed a higher amount of gentamicin loading than that in 5:5 and 6:4 PLA for a long duration of 168 h, maintaining a constant release concentration of 4.23 µg mL^−1^. For 5:5 PLA, gentamicin release stopped after about 72 h reaching a constant level that was same as the control. Drug release mechanisms are related to diffusion [34]. The initial release involves the rapid diffusion of antibiotics deposited on the fiber surface, while the slow release mainly includes the diffusion of antibiotics through the polymer matrix [35]. Interconnected porous 7:3 PLA structures can provide additional sites for adsorption of gentamicin and enable the slow and continuous release of the drug owing to the high specific surface areas, pore-volume, and porosity. In this study, 7:3 PLA had the highest drug loading and release rate among all fibers. Cumulative dentamicin release patterns are influenced by the morphology of porous PLA fibers. The release profile of gentamicin-loaded PLA samples was generated in vitro for 7 d (168 h) in Figure 7b. The cumulative amount of gentamicin delivered by porous PLA fibers during 7 d was much higher than that by nonporous PLA, as indicated by 7:3 PLA > 6:4 PLA > 5:5 PLA > non-porous PLA. As a result, gentamicin release continued for 168 h. All PLA samples at the initial stage displayed burst release, which is favorable for the rapid relief of bacterial infection. However, a favorable release system should not only meet the gentamicin release capacity but also be able to control the release rate [13]. Therefore, 7:3 PLA emerged as a promising antibiotic carrier that can facilitate the continuous release of gentamicin over a long period.

## 4. Conclusions

Overall, our results showed that solvent ratios of DCM/AC in polymer–solvent systems have a significant effect on the morphology of porous PLA fibers in terms of pore size, volume, and specific surface area. As a result, an increase in the quantity of DCM from 50 to 70% in polymer–solvent systems led to an increase in average fiber diameter from 1.87 to 2.43 µm, formation of a more porous interconnected structure, and increased specific surface area (55.61 m^2^ g^−1^), pore size (42.19 nm), and pore volume (12.78 cm^3^ g^−1^). Antibacterial properties of AD-loaded 7:3 PLA fibers were evaluated against *E. coli* and *S. epidermidis*. For all AD-loaded 7:3 PLA fibers against *S. epidermidis*, the inhibition effect was reduced after 24 h of incubation, whereas its antibacterial activity against *E. coli* increased or remained constant for 72 h. Furthermore, kanamycin, gentamicin, or amikacin-loaded 7:3 PLA have been evaluated for their antibacterial properties by kinetic growth curve tests. The antibacterial activity was as follows: gentamicin > amikacin > kanamycin, which could be due to gentamicin having the highest electrostatic binding energy to the negatively charged bacterial membrane.

Gentamicin release patterns according to the PLA fibers (porous 7:3, 6:4, 5:5, and non-porous PLA fibers) were evaluated for 168 h. Interconnected porous 7:3 PLA can carry the highest amount of gentamicin by providing additional sites for adsorption and enabling the slow and continuous release of the drug, thereby optimizing the treatment of patients and having long-term uses. Further detailed study on the release profile of other ADs such as kanamycin and amikacin is required. This study helps to better understand the formation of porous PLA fibers by electrospinning and demonstrates the fiber morphology-antibacterial activity relationship. Additionally, this study may provide the time design for the rapid or long-term relief of bacterial infection.

## Figures and Tables

**Table 1:** Physical properties of electrospun non-porous and porous PLA fibers.

Polymer–Solvent System (Name Code)	Average Fiber Diameter (µm)	Average Pore Diameter (nm)	Specific Surface Area (m^2^ g^−1^)	Total Pore Volume (cm^3^ g^−1^)	Viscosity (mPa·s)
5:5 DCM: AC porous PLA (5:5 PLA)	1.866 (±0.56)	19.23 (±3.26)	5.877 (±0.82)	0.145 (±0.01)	193 (±2.48)
6:4 DCM:AC porous PLA (6:4 PLA)	1.816 (±0.58)	30.29 (±4.12)	28.96 (±1.14)	6.659 (±0.29)	218 (±4.02)
7:3 DCM:AC porous PLA (7:3 PLA)	2.434 (±0.85)	42.19 (±4.85)	55.61 (±3.08)	12.78 (±0.46)	252 (±3.86)
7:3 CF:EtOH non-porous PLA (non-porous PLA)	2.280 (±1.11)	-	0.030 (±0.04)	0.0009 (±0.0001)	292 (±6.16)

**Table 2:** Inhibition (%) of AD-loaded porous 7:3 PLA and free ADs against *E. coli* and *S. epidermidis* after 72 h.

Bacteria Stain	Gentamicin-7:3 PLA	Kanamycin-7:3 PLA	Amikacin-7:3 PLA	Free Gentamicin	Free Kanamycin	Free Amikacin
*E. coli*	83 (±3.8)	46 (±0.8)	85 (±1.1)	53 (±3.7)	40 (±2.8)	55 (±1.6)
*S. epidemidis*	93 (±2.7)	22 (±6.3)	16 (±4.2)	47 (±1.7)	18 (±2.9)	8 (±1.8)

## Data Availability

The data presented in this study are available from the corresponding author upon reasonable request.

## Supplementary Materials

The following supporting information can be downloaded at: https://www.mdpi.com/article/10.3390/pharmaceutics14061272/s1, Figure S1: Antibacterial activities of free gentamicin, kanamycin, and amikacin for 72 h; Figure S2: Gentamicin concentration-OD_405_ standard curve.
