# Supplementary material for: Exploring the Diverse Morphology of Porous Poly(Lactic Acid) Fibers for Developing Long-Term Controlled Antibiotic Delivery Systems
Source: Pharmaceutics. 2022 Jun 15;14(6):1272. doi: 10.3390/pharmaceutics14061272 (PMC9231122; doi:10.3390/pharmaceutics14061272)
Supplement: Supplementary file 1 [file pharmaceutics-14-01272-s001.zip › pharmaceutics-1706604-supplementary.pdf]

Supporting information

Exploring the diverse morphology of porous poly(lactic acid) fibers for developing long-term controlled antibiotic delivery systems

Juran Kim[1*] and Kwon Ho Seo[1], Kyung Eun Lee[2] and Meltem Yanilmaz[3*]

[1]Advanced Textile R&D Department, Korea Institute of Industrial Technology (KITECH), Ansan 15588, Korea; tjrnjsgh@kitech.re.kr (K.H.S.)

[2]Department of Mechanical Engineering, Inha University, 100 Inharo, Incheon, 22212, Republic of Korea; bfmec@inha.ac.kr (K.E.L)

[3]Textile Engineering, Istanbul Technical University, 34467 Istanbul, Turkey; *yanilmaz@itu.edu.tr* (M.Y)

*Correspondence: jkim0106@kitech.re.kr (J. K.)

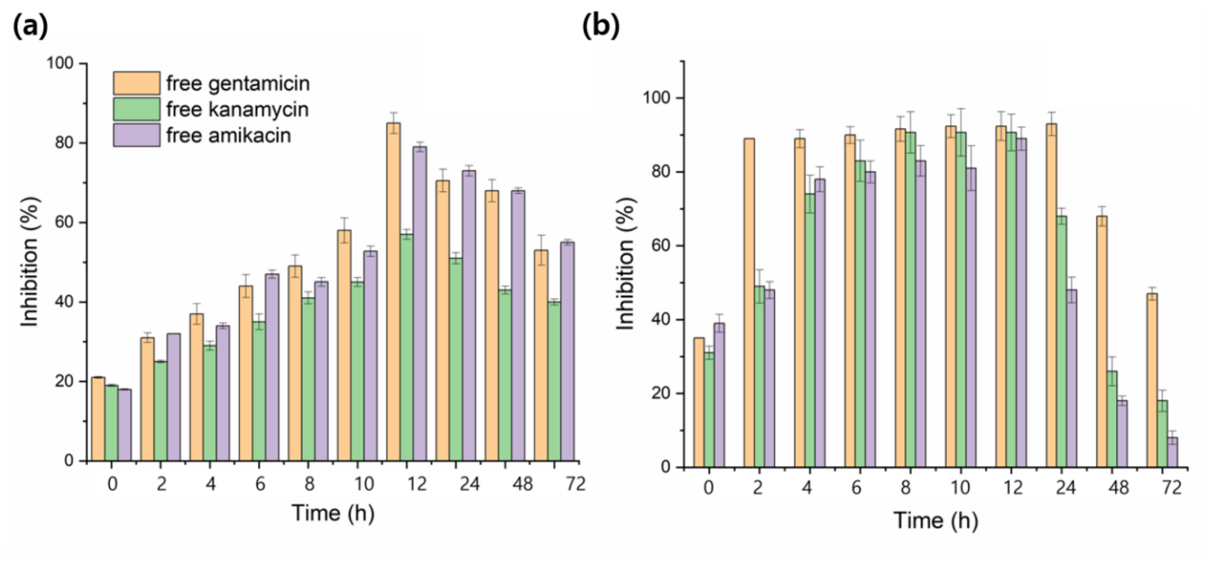

Figure S1. Antibacterial activities of free gentamicin, kanamycin, and amikacin for 72 h.

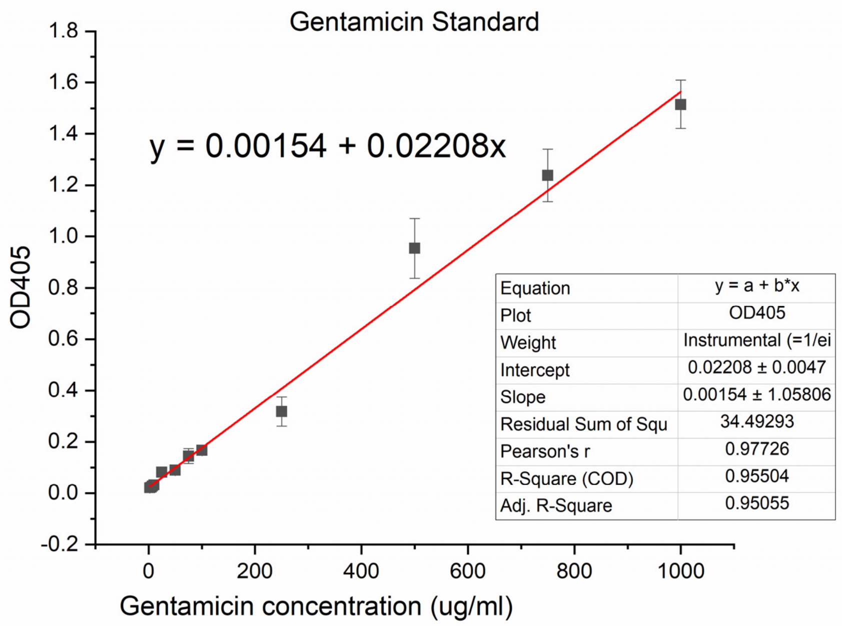

Figure S2. Gentamicin concentration-OD_{405} standard curve.
